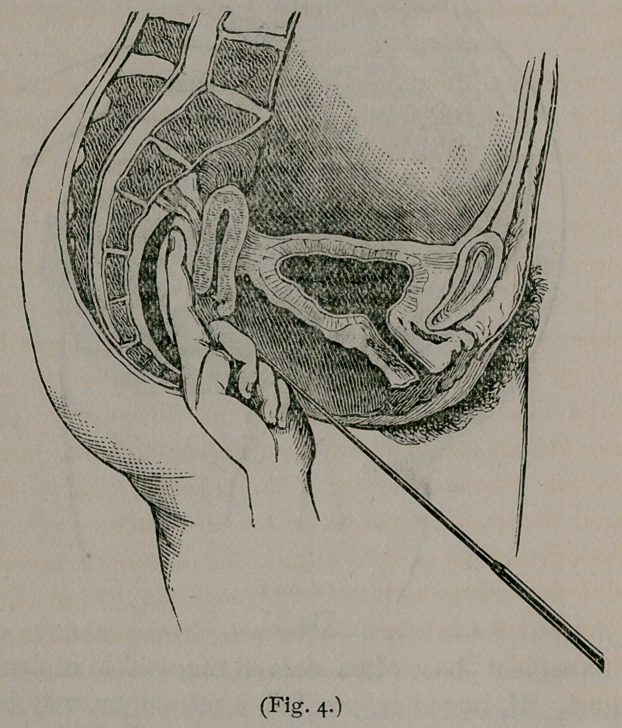# A Systematic Stretching for Shortening of the Broad and Utero-Sacral Ligaments

**Published:** 1888-04

**Authors:** Geo. H. Noble

**Affiliations:** Atlanta, Ga.


### A SYSTEMATIC STRETCHING FOR SHORTENING OF THE BROAD AND UTERO-SACRAL LIGAMENTS.*

BY GEO. H. NOBLE, M. D., ATLANTA, GA.

For the past four years I have been endeavoring to relieve that condition known as shortening of the broad and utero-sacral ligaments by stretching, massage, etc., and having arrived at a degree of success I am now constrained to present it to the profession. My silence upon this subject is due to the fact that I did not desire to conjecture, but to wait and watch for results.

The condition of the structures we have to deal with is contraction after cellulitis, generally complicated by some disease of other pelvic organ or organs. *Apparently* there is no loss of tissue, but a shrinking in the length and an increase in the thickness of the ligament, the effect of which is most disastrous to the comfort and well-being of the patient. The diseased tissue, acting from a fixed point upon the bony wall of the pelvis, draws the uterus in that direction and necessarily takes upon itself the greater part of the weight of the uterus.[a] It also exerts an abnormal degree of traction upon the opposite ligament and greatly modifies the mobility of the uterus; when, therefore, a cough or step gives the organ a quick, downward motion its force is expended upon the diseased ligament. This repeated day after day keeps up an irritation which adds to the weight of the uterus by setting up congestive hyperplasia and other complications. The woman becomes an invalid for the want of exercise, and the case goes on from bad to worse until an attack of cellulitis is developed in the othe1 ligament.—(Emmett.)

If, however, the inflammation has been confined to the lower portion of a broad ligament, the cervix only may be displaced[b],

*This paper was prepared for the Georgia State Medical Association in February, 1887, but owing to my absence from the State it was overlooked.

[a]Illustrated in Emmett's Gynæcology.

[b]The uterus may sometimes be congenitally placed a little to one side.

and the case pursue a course not so well marked, but often distressing from vesicle tenesmus set up by constant dragging upon the base and neck of the bladder.

When the utero-sacral ligaments have been the seat of inflammation, the contraction draws the uterus backward and a little upward and renders tense the utero-vesicle attachments, which in marked cases may feel like cords to the finger. Not unfrequently the cavity of the bladder is by this means transformed into an exaggerated triangular space, with its base at the symphysis pubis, and apex at the uterine attachment. When this occurs it is often impossible to voluntarily expel its full contents, as the tense walls will not allow the bladder to contract sufficiently to completely drive out the urine, the remaining portion of which may decompose and add greatly to the irritation.

The examining finger will detect the uterus resting upon a line running from the neck of the bladder to the sacral attachments of the utero-sacral ligaments, which is formed in part by the diseased ligaments and in part by the base of the bladder. Therefore the force applied to the uterus in its downward motion is expended upon this line, and as the diseased ligaments are inelastic, the base and neck of the bladder must receive the brunt of the blow. This keeps up an almost constant jerking and tugging at the base of the bladder, and excites thickening of the walls of that organ, which, if left to run its course, will end in fatal disease of the kidney.

The foregoing is but a brief outline merely presented to illustrate some of the causes and effects.

Now, the main feature in my treatment is a systematized stretching conjoined, when practicable, with such manipulations as are advised by Dr. Van de Warker for adhesions and thickening in the pelvis—notably by rubbing or rolling of the thickened parts between the fingers of the two hands. This latter is often difficult and impracticable, but should not be neglected when applicable.

The first indications are to relieve the pelvic tenderness and to free the ligaments of all sources of irritation. To do this the weight of the uterus must be removed from the ligaments by a non-irritating support, such as the wool or cotton and glycerine tampon, observing, however, the precautions not to use too large a dressing, as it will work discomfort, or even detriment, by forcing the uterus too high in the pelvis and putting the ligaments upon the stretch just at a time when they should have rest. Then when the parts are in good condition, the manipulations designed for the elongation of the ligaments may be commenced.

The operator, in treating the broad ligament, should use the hand opposite to the side in which the disease is situated. For instance, if it is the left broad ligament, two fingers of the right-hand should be introduced and placed on the left side of the cervix. Then the fingers of the other hand are brought down from above the pelvic brim by depressing the abdominal walls, and are placed on the same side of the uterus at the fundus. (See fig. 1).

(Fig. 1.)

Very gentle traction in the direction of the healthy ligament is then made and maintained from a few moments to a number of minutes (5 or 20), according to the degree of discomfort and tendency to inflammation.

For shortening of the utero-sacral ligaments the two first fingers of the hand most accustomed to vaginal examinations should be introduced and placed posterior to the cervix. The finger-tips of the other hand then depress the abdominal wall and are applied to the posterior surface of the fundus uteri. (See fig. 2.)

(Fig. 2.)

The uterus is then gently drawn forward and held in that position for some minutes, as above stated. A small tampon, sufficient to take the weight of the uterus, should be applied after each operation. In extreme contractions, the uterus may be drawn back to the sacrum, and considerable difficulty will be experienced in grasping it, especially if there be much fat or a rigid condition of the rectii muscles; but persistence in steady pressure may overcome the trouble by gaining a little advancement at each expiration until the fingers reach the promontory of the sacrum. Then, by following down the surface of that bone, you may finally reach the uterus. But if you are unable to insert the fingers between the uterus and the sacrum, remove the fingers from the cervix and press the other hand deeper into the hollow of the sacrum, and while holding it in this position, force the uterus directly upward with the fingers in the vagina until it rises well upon the hand above. (Represented by dotted lines in fig. 3.) In this way you may gain perfect control of the organ.

(Fig. 3.)

This expedient has often served me well in replacing retroverted uteri. If, however, you fail, a tenaculum may be hooked in the posterior lip and the uterus drawn a little downward and forward, while the index finger passes high up in the posterior cul-de-sac, and makes pressure forward near the fundus. (See fig. 4.) This is especially serviceable when the uterus is flaccid. If preferred, the finger may be passed into the rectum and pressure made on the uterus in the direction of the symphysis pubis.

Adhesions are not uncommon complications, and are to be treated the same way, except when the uterus is adhered directly to the rectum. In such cases, attempts at stretching the attachments generally fail, as the rectum is not a fixed point, and any effort at stretching will pull the rectum in the direction in which the traction is made.

In a case of shortening of the utero-sacral ligaments, with extensive adhesions to the sacrum just below the promontory, and in which I utterly failed to grasp the uterus with my fingers, I stretched the posterior cul-de-sac with my forceps, protected at the point with a small ball of cotton, until firm pressure could be

(Fig. 4.)

brought to bear upon the posterior surface of the fundus, after which it became an easy matter to stretch up the adhesions. But upon two occasions my forceps slipped from the back to the side of the uterus with such suddenness that for the time I was filled with alarm, and thought that I had torn the uterus away from the sacrum.

Now two questions will naturally arise, viz.: How hard shall we pull upon the ligaments, and how much should they be stretched? The first is about as difficult to answer as it is to say how hard we should pull upon the obstetrical forceps. But the rule that I always observe in the beginning of the treatment (and it is one that confines the manipulations entirely within the bounds of safety) is to make just enough traction to feel that you are barely putting the tissue upon the stretch. This is slightly increased at each seance until I learn how much each case can stand. You should bear in mind, however, that if the manipulations are too vigorous the parts will become tender, which, when it occurs, is an indication for the suspension of the process until the soreness is removed.

As to the second query, I should say that if you desire permanent results you should endeavor to stretch the ligament about twice as much as it has been contracted—that is, the treatment should be continued until the ligament or ligaments will allow the uterus to be carried as much beyond the centre or median line of the pelvis as it has been drawn away from that point. For instance, a case had the uterus drawn so close to the right side of the pelvis that I could not insert my finger between it and the bone, but by continued treatment I was enabled to carry the uterus with ease to the opposite side of the pelvis. Again the uterus was drawn down to the sacrum by diseased utero-sacral ligaments, but was so relieved of its incarceration by stretching that it could be drawn forward to the ossa pubis.

You should endeavor to elongate the ligament to that extent which will leave behind it the greatest physiological mobility of the uterus after some secondary shortening has taken place; otherwise, you do not effect a cure, even though your patient may be comfortable.

The time consumed in this treatment varies greatly. A few weeks may suffice to relieve the more urgent symptoms in slight contractions, but if it is suspended at this stage the results will not be lasting, for we lose a certain amount of the benefits in the secondary contractions. To secure permanent results in extreme cases, a number of months, or even a year and a half, may be required. There are several reasons for this: 1st. The extent of the disease is greater. 2d. The extent of the stretching must be greater; and 3d, there is less healthy tissue in the ligaments to stretch.

Below I give a short summary of cases in private practice. No clinical cases are recorded, as they were lost sight of:

5 had shortening of left broad ligament.

3 had shortening of right broad ligament.

4 had shortening of utero-sacral ligaments.

2 had shortening of left broad ligament and utero-sacral ligaments, and a number of others left before the treatment was completed, claiming an inability to meet expenses, while some others are now under treatment.

## Figures and Tables

**Fig. 1. f1:**
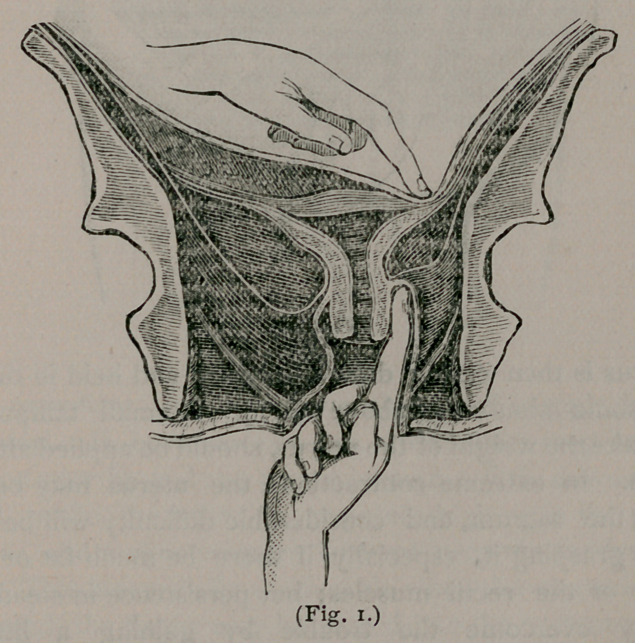


**Fig. 2. f2:**
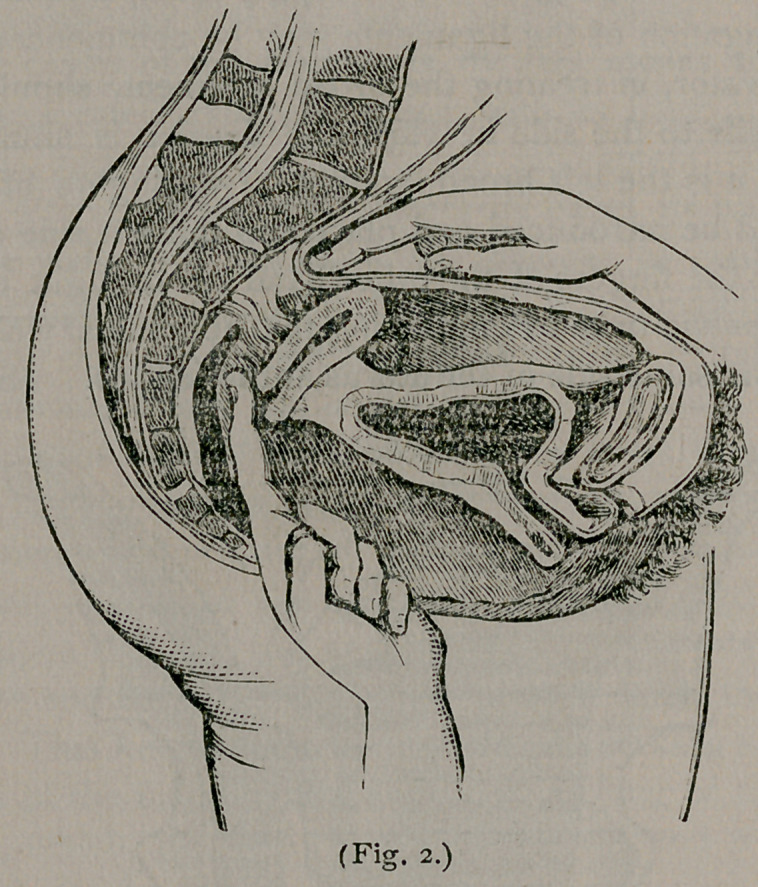


**Fig. 3. f3:**
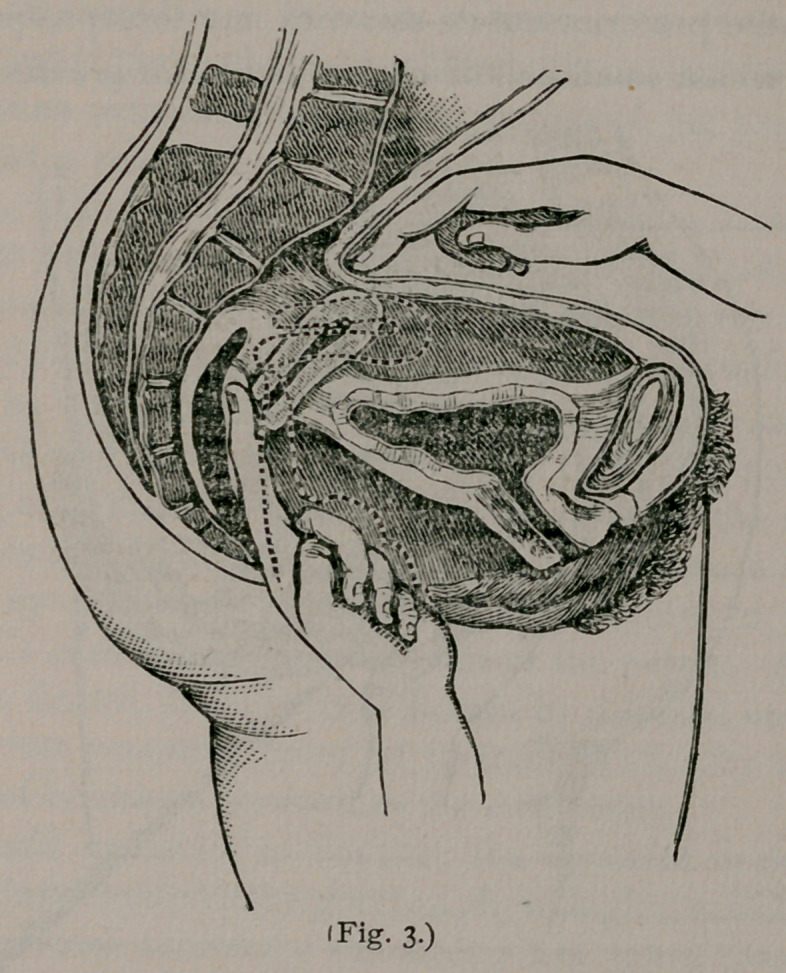


**Fig. 4. f4:**